# Treatment of Osteoporosis in Chronic Kidney Disease

**DOI:** 10.1590/2175-8239-JBN-2021-S109

**Published:** 2021-12-03

**Authors:** Fellype Carvalho Barreto, Sérgio Gardano Elias Bucharles, Vanda Jorgetti

**Affiliations:** 1 Universidade Federal do Paraná, Internal Medicine Department, Service of Nephrology, Curitiba, PR, Brazil.; 2 Universidade Federal do Paraná, Hospital de Clínicas, Service of Nephrology, Curitiba, PR, Brazil; 3 Universidade de São Paulo, Faculdade de Medicina, Hospital das Clínicas, Pathophysiology Laboratory (LIM-16), São Paulo, SP, Brazil.

## Recommendations

1. For CKD G1-2 patients with osteoporosis, fragility fractures or high risk for fractures, the treatment should be similar to the one offered to the general population (Evidence).

2. For CKD G3a-b patients with osteoporosis, fragility fractures or high risk for fractures, without biochemical alterations of CKD-mineral and bone disorder (CKD-MBD), the treatment should be similar to that given to the general population (Evidence).

3. For CKD G3a-5D patients with osteoporosis, fragility fractures or high risk for fractures and with biochemical alterations of CKD-MBD, the treatment of CKD-MBD should be optimized before initiation of therapy for osteoporosis (Opinion).

4. For CKD G4-5D patients, any anti-osteoporotic pharmacological treatment, whether antiresorptive or anabolic, is empirical, given the low clinical evidence. (Opinion)

4.1 Although not mandatory, bone biopsy should be considered before starting treatment with anti-osteoporotic drugs. (Opinion) 

5. For CKD G1-G5D patients, non-pharmacological interventions should be considered, including smoking cessation, moderate alcohol intake, increased physical activity, and fall prevention (Opinion).

6. For CKD G1-5D patients receiving anti-osteoporotic therapy, a 1-2 year DEXA interval is suggested (Opinion).

## Rational

In patients with chronic kidney disease (CKD), the prevalence of osteoporosis and fragility fractures is significantly higher than in the general population,^1^ ^,^ ^2^ ^,^ ^3^ ^,^ ^4^ resulting in impaired quality of life and increased morbidity and mortality. The pathophysiology of bone disease in the CKD setting is complex and still not fully elucidated, and its treatment is a real challenge^5^. The risk of fracture increases as the renal function declines. The cumulative incidence of fractures over 3 years is about 5% in men and almost 10% in women over 65 years old and estimated glomerula filtration rate (eGFR) < 15 mL/min/1.73m^2^, while for patients in the same age group and with a eGFR > 60 mL/min/1.73m^2^, it is 1.6% for men and 4.3% for women^6^. Among NHANES III study participants with CKD, the prevalence of fractures was twice as high as that observed among participants without CKD^7^. Furthermore, in the chronic dialysis population, patients who had hip fracture had a 50% shorter mean survival when compared to patients matched and controlled for the presence of cardiovascular disease, age, and dialysis vintage, but without fractures^8^. 

The World Health Organization (WHO) defines osteoporosis as a progressive systemic skeletal disease characterized by low bone mass, microarchitectural deterioration, with consequent increased fragility and risk of fracture^9^. While bone mass may be assessed by two-dimensional (dual-energy absorptiometry - bone densitometry, DEXA) or three-dimensional (peripheral computed tomography) radiological examinations^5^, bone quality, of which the main components are turnover, mineralization, collagen structure, and microarchitecture, is best assessed by biopsy and histomorphometric analysis^10^. 

Both cortical and trabecular bone are responsible for bone strength, being approximately 80% of the skeleton composed of cortical bone. Disorders of mineral and bone metabolism in CKD significantly contribute to reduced quality of this tissue. Trabecular bone volume may be decreased in any pattern of renal osteodystrophy^11^. Secondary hyperparathyroidism (SHPT) may lead to increased porosity and reduced cortical thickness throughout the various stages of CKD^12^ ^-^ ^15^. 

Additionally, advanced age, hypogonadism, and the use of certain drugs (corticosteroids and calcineurin inhibitors) could result in trabecular bone loss, associated or not with mineralization defects^5^. Other contributors to bone quality loss include oxidative stress, the accumulation of advanced glycation end products, as well as malnutrition, metabolic acidosis, diabetes mellitus, and hypovitaminosis D^5^. 

Although bone biopsy represents the "gold standard" for diagnosing the type of renal osteodystrophy, the most recent international guidelines on CKD-MBD do not recommend its mandatory performance before starting osteoporosis treatment, recognizing the difficulties in obtaining and analyzing it. It is suggested that PTH and alkaline phosphatase dosages may be used to assess the possible type of bone turnover, since markedly low or high values of these biomarkers reflect low and high turnover bone disease, respectively^16^ ^,^ ^17^. Bone biopsy should be reserved for cases in which the diagnosis of the type of renal osteodysthrophy is not clear, what could help in choosing the anti-osteoporotic treatment^16^. 

## Treatment of osteoporosis associated with chronic kidney disease

### General Aspects

Control of traditional risk factors linked to osteoporosis and fragility fractures should be encouraged. Although there are no randomized clinical trials in CKD patients, they should be stimulated to perform physical activity^18^ and to take fall prevention measures^19^, in order to reduce the risk of fragility fractures.

The first line of specific care in the treatment of CKD-associated OP is the control of CKD-MBD, which should be managed before the initiation of usual pharmacotherapy for osteoporosis^16^. The aim is to maintain calcium and phosphate serum levels within the normal range, a suggestion to keep PTH within the normal range in CKD G3-G5, and between 2 and 9 times the upper limit of the method in CKD G5D. Although there is no target range of PTH values that unequivocally results in reduced risk of fracture, Limori et al., in a retrospective study, found that serum levels of PTH between 150-300 pg/mL were associated with lower risk of fracture when compared to PTH outside this range in hemodialysis patients ^20^. 

With regard to calcium and vitamin D supplementation, it is known that long-term calcium deficiency is associated with an increased risk of osteoporosis. However, there is little evidence that calcium supplementation prevents fractures^21^. In addition, some studies have suggested that the use of compounds containing calcium salts might be associated with cardiovascular events ^22^. Particularly in CKD patients, positive calcium balance may be deleterious due to the risk of ectopic calcification ^23^. It is important to mention that the use of ergo or cholecalciferol, especially the latter compound, to correct hypovitaminosis D might contribute to a reduction in the risk of falls^24^, and that the deleterious effects of osteomalacia secondary to hypovitaminosis D should not be ignored^25^. Daily doses of at least 800 IU of vitamin D are associated with reduced risk of fractures in elderly patients^24^. Although optimal vitamin D levels are not well established for CKD patients, some studies suggest that levels > 30 ng/mL may be satisfactory^26^. 

### Use of antiresorptive drugs

*Bisphosphonates*

They are inorganic pyrophosphate analogs, with high affinity for bone mineral matrix, that inhibit osteoclast-mediated bone resorption^27^. Nitrogen-containing bisphosphonates (alendronate, risedronate, ibandronate, pamidronate, and zoledronic acid) inhibit the enzyme farnesyl pyrophosphatase synthase, a key process in the mevalonate pathway, inducing osteoclast apoptosis, while preserving osteoblast and osteocyte activity^28^ ^,^ ^29^. Bisphosphonates are well established as the first-line treatment for several types of osteoporosis (juvenile, postmenopausal, senile, immobility-induced)^30^. As these medications are excreted by the kidneys and have long half-lives, they should be avoided in patients with eGFR < 30 mL/min/1.73m^2^.^30^

International guidelines suggest that patients in CKD G1-G3 with osteoporosis and/or high risk of fragility fractures, with controlled CKD-MBD, should be treated similarly to the general population^16^. International guidelines for the general population suggest that therapy with oral bisphosphonate should not exceed 5 years in patients with densitometric criteria for osteoporosis and/or fragility fractures, or else in patients with a ≥ 3% probability of major osteoporotic fracture within 10 years^9^. This is not, however, an absolutely established norm. More recent studies suggest that those patients may benefit from its use for up to 10 years, with periodic assessments of the potential benefits and risks of the medication^31^.

Besides the potential side effects in the gastrointestinal tract (gastroesophageal reflux, esophagitis), muscle pain, uveitis, hypocalcemia, and fever (injectable forms), there are two major concerns: osteonecrosis of the jaw and low turnover bone disease^30^. The risk of osteonecrosis of the jaw increases after exposure to high doses of injectable bisphosphonates and appears to be much less frequent with oral formulation. Careful dental hygiene and regular dental treatment are of paramount importance^27^. Low turnover bone disease, characterized by suppression of bone turnover, does not seem to be a frequent complication bisphosphonate therapy in the general population^32^. 

*Post hoc* analyses of large randomized trials assessing the safety and efficacy of bisphosphonate for the treatment of menopause-related osteoporosis have demonstrated that these drugs (alendronate and risedronate) have comparable efficacy in bone mass recovery at femoral neck and lumbar spine, in addition to prevention of risk of vertebral fractures, among women with CKD G3-G4^33^ ^,^ ^34^. Bone biopsies, performed on a few patients who participated in these studies, did not reveal low turnover bone disease or mineralization defects. More recently, other studies in populations at different stages of CKD (G2, G3a and G3b) demonstrated that risendronate was safe and induced a significant bone mass gain and reduction in the levels of bone turnover biomarkers (N-terminal telopeptide of type I collagen, C-terminal telopeptide of type I collagen, and bone fraction of alkaline phosphatase), similar to that observed in patients without CKD (eGFR > 90 mL/min/1.73m^2^)^35^ ^,^ ^36^, without altering renal function^35^. It should be mentioned that the aforementioned studies included patients with CKD G2-G4, with no evidence of CKD-MBD. 

Few studies have evaluated the use of bisphosphonates in patients with advanced CKD and CKD-MBD. Toussaint et al. reported an increase in bone mineral density of the lumbar spine over a period of 18 months in patients with CKD G3 and G4 treated with alendronate compared with placebo ^37^. Bergner et al. demonstrated in a 48-week study, which included 16 dialysis patients with osteopenia and hyperparathyroidism, that ibandronate led to an increase in the lumbar spine bone mass, without changing significantly the PTH serum values. None of these studies assessed bone histomorphometric analysis before or after treatment, in order to obtain information on bone turnover. Ota et al, in an animal model of late-stage CKD, observed that alendronate improved the trabecular bone volume and mineralization, without affecting residual renal function^39^.

Few studies have evaluated the clearance of bisphosphonates by dialysis. Bergner et al. observed in 12 stable patients on hemodialysis that about 36% of total sodium ibandronate intravenously administered was removed after the first hemodialysis session, while the plasma concentration of the drug in relation to its maximum peak was reduced by 78% after a 4-hour hemodialysis session^40^. Iseri et al. studied 6 osteoporotic patients on chronic hemodialysis and found that the intradialytic clearance of intravenous alendronate sodium is approximately 50% of, a clearance similar to that observed in patients with preserved renal function^41^.

*Denosumab*

Denosumab is a human monoclonal antibody that targets the receptor activator of nuclear factor-kappa B ligand (RANKL). It blocks the binding of RANKL to its receptor (RANK), which reduces osteoclastic activity, bone resorption and formation. The excretion and metabolism of denosumab do not depend on the renal system, occurring via the reticuloendothelial system. It is administered subcutaneously, every 6 months. The use of denosumab for 36 months in postmenopausal women with osteoporosis significantly increased bone mineral density in spine, hip and radius and decreased the risk of vertebral and non-vertebral fractures^42^. 

There are no randomized, placebo-controlled clinical trials designed specifically to assess the effects of denosumab in the CKD population. A *post hoc* analysis of the "Fracture reduction evaluation of denosumab in osteoporosis every 6 months (FREEDOM)" study demonstrated that in patients (N = 2890) with CKD and eGFR < 60 mL/min/1.73m^2^, by the Cockcrof-Gault formula, denosumab reduced the incidence of vertebral fractures and increased bone mineral density at all sites (lumbar spine, femoral neck, total hip) during the 36-month study period, regardless of renal function stage. No significant reduction in the number of non-vertebral fractures was noticed. Adverse effects were similar between CKD and non-CKD patients, and there was no effect on renal function^43^. It is important to note that: (i) most participants had CKD G3 (N = 2817), only a minority had CKD G4 (N = 73), (ii) none of them had hyperparathyroidism or hypocalcemia, CKD-MBD biochemical abnormalities commonly present in these stages of CKD, as they were part of the exclusion criteria, and (iii) the formula used to estimate GFR is not considered to have the highest accuracy. 

Prospective, uncontrolled, short-term studies with a small number of patients have reported the beneficial effect of denosumab on bone mass in hemodialysis patients. Although these results may be encouraging, it is worth mentioning that none of these studies reported an effect on the incidence of fracture. An increased incidence of hypocalcemia, both symptomatic and asymptomatic, was observed, especially on the seventh day after medication administration. This change may be satisfactorily managed with calcitriol dose adjustment, calcium supplementation, or increasing the calcium concentration in the dialysate. ^44^ ^-^ ^46^. Finally, there are no studies evaluating long-term safety of the drug neither in stages G4, G5 and G5D CKD patients, nor in peritoneal dialysis patients. 

### Use of anabolic drugs

*Teriparatide*

Teriparatide is a recombinant peptide containing the first 34 amino acids of human PTH. There are two presentations with different doses and frequency of subcutaneous administration, one daily (20 mcg) and the other weekly (56.5 mcg). 

In a *post hoc* analysis of the Fracture Prevention Trial, teriparatide, at a dose of 20 or 40 mcg/day, in patients with mild (eGFR between 50-79 mL/min/1.73m^2^) or moderate (eGFR between 30-49 mL/min/1.73m^2^) CKD, was associated with bone mass gain in the lumbar spine and femoral neck, and reduced the risk of vertebral and non-vertebral fracture, at a median follow-up time of 19 months.^47^ A higher incidence of hypercalcemia and hyperuricemia was observed in patients with kidney dysfunction compared to those with normal kidney function, with no evidence of increased risk for gout, arthralgia, or nephrolithiasis^47^.

Only uncontrolled studies with a small number of patients have evaluated teriparatide in the treatment of osteoporosis in hemodialysis patients with relatively low PTH levels^48^ ^,^ ^49^. The use of teriparatide, at a dose of 56.5 mcg/week for 1 year, was associated with bone mass gain in the lumbar spine. It is worth mentioning the occurrence of an elevated number of adverse effects, mainly hypotension, which led some patients to discontinue the medication ^49^. A study evaluating teriparatide (20 mcg/day) in 8 hemodialysis patients with adynamic bone disease reported bone mass gain in lumbar spine and femoral neck, although without reaching significance, which could be explained by the small number of patients evaluated^50^. There are no studies evaluating neither the use of teriparatide in peritoneal dialysis patients nor its long-term efficacy and safety in dialysis patients.
